# Measuring Bound Attention During Complex Liver Surgery Planning: Feasibility Study

**DOI:** 10.2196/62740

**Published:** 2025-01-08

**Authors:** Tim Schneider, Timur Cetin, Stefan Uppenkamp, Dirk Weyhe, Thomas Muender, Anke V Reinschluessel, Daniela Salzmann, Verena Uslar

**Affiliations:** 1 University Hospital for Visceral Surgery, PIUS-Hospital Department for Human Medicine, Faculty VI University of Oldenburg Oldenburg Germany; 2 Department of Medical Physics and Acoustics Faculty VI University of Oldenburg Oldenburg Germany; 3 Digital Media Lab Department of Mathematics and Computer Science, Faculty III University of Bremen Bremen Germany; 4 Department of Computer and Information Science University of Konstanz Konstanz Germany

**Keywords:** workload measurement, virtual reality, VR, augmented reality, AR, electroencephalography, EEG, event-related potential, ERP, auditory evoked potential, AEP, oddball experiment, National Aeronautics and Space Administration Task Load Index, NASA-TLX, surgical planning

## Abstract

**Background:**

The integration of advanced technologies such as augmented reality (AR) and virtual reality (VR) into surgical procedures has garnered significant attention. However, the introduction of these innovations requires thorough evaluation in the context of human-machine interaction. Despite their potential benefits, new technologies can complicate surgical tasks and increase the cognitive load on surgeons, potentially offsetting their intended advantages. It is crucial to evaluate these technologies not only for their functional improvements but also for their impact on the surgeon’s workload in clinical settings. A surgical team today must increasingly navigate advanced technologies such as AR and VR, aiming to reduce surgical trauma and enhance patient safety. However, each innovation needs to be evaluated in terms of human-machine interaction. Even if an innovation appears to bring advancements to the field it is applied in, it may complicate the work and increase the surgeon’s workload rather than benefiting the surgeon.

**Objective:**

This study aims to establish a method for objectively determining the additional workload generated using AR or VR glasses in a clinical context for the first time.

**Methods:**

Electroencephalography (EEG) signals were recorded using a passive auditory oddball paradigm while 9 participants performed surgical planning for liver resection across 3 different conditions: (1) using AR glasses, (2) VR glasses, and (3) the conventional planning software on a computer.

**Results:**

The electrophysiological results, that is, the potentials evoked by the auditory stimulus, were compared with the subjectively perceived stress of the participants, as determined by the National Aeronautics and Space Administration-Task Load Index (NASA-TLX) questionnaire. The AR condition had the highest scores for mental demand (median 75, IQR 70-85), effort (median 55, IQR 30-65), and frustration (median 40, IQR 15-75) compared with the VR and PC conditions. The analysis of the EEG revealed a trend toward a lower amplitude of the N1 component as well as for the P3 component at the central electrodes in the AR condition, suggesting a higher workload for participants when using AR glasses. In addition, EEG components in the VR condition did not reveal any noticeable differences compared with the EEG components in the conventional planning condition. For the P1 component, the VR condition elicited significantly earlier latencies at the Fz electrode (mean 75.3 ms, SD 25.8 ms) compared with the PC condition (mean 99.4 ms, SD 28.6 ms).

**Conclusions:**

The results suggest a lower stress level when using VR glasses compared with AR glasses, likely due to the 3D visualization of the liver model. Additionally, the alignment between subjectively determined results and objectively determined results confirms the validity of the study design applied in this research.

## Introduction

In modern surgery, new techniques are increasingly being adopted. One example of such innovative technologies is minimally invasive procedures, which aim to reduce surgical trauma and enhance patient safety [1]. However, while these techniques benefit the patient, they pose significant challenges—both cognitively and physically—for the surgical team, particularly the surgeon, due to limited visibility and increased physical strain. This, in turn, may jeopardize patient safety [2].

Another example of novel technologies applied in surgery is the use of augmented reality (AR) and virtual reality (VR) simulations. These simulations display 3D models of anatomical structures on a 3D screen, potentially aiding in their examination and analysis. Glasses that project these simulations can be used during the preoperative phase to assist with surgical planning.

To incorporate technologies such as AR and VR applications into everyday clinical practice or the operating room, these technologies must first be evaluated for safety, ergonomics, and particularly for their impact on workplace strain [3]. So far, AR and VR glasses have primarily been assessed for mental workload using subjective questionnaires, such as the National Aeronautics and Space Administration-Task Load Index (NASA-TLX), or by measuring the user’s performance quality [4]. This study aims to assess mental workload both objectively, through electrophysiological recordings, and subjectively, using established questionnaires, to facilitate a comparison between the 2. The study is not intended to evaluate or rate the different techniques used.

From a neuroergonomic perspective, replacing subjective questionnaires with objective measurements offers distinct advantages. Subjective questionnaires rely on self-reporting, which can be influenced by various biases. By contrast, objective measurements provide more accurate and reliable data by directly assessing neurophysiological and behavioral responses in real time [5]. By minimizing reliance on subjective questionnaires, objective measurements enhance the scientific validity of neuroergonomic research, resulting in more robust findings and practical implications that could optimize human-machine interactions.

AR and VR glasses enable more accurate planning of invasive procedures through 3D visualization of anatomical structures. This allows the medical team to anticipate potential anomalies and develop a tailored plan for complex surgeries that considers the patient’s individual anatomy more precisely than traditional preparation using conventional radiological images. Such technologies are particularly valuable in challenging interventions with limited direct visibility, such as laparoscopic procedures [6]. Additionally, VR and AR can serve as effective training tools for inexperienced surgeons, helping them gain proficiency and improve safety during interventions.

Several studies have subjectively evaluated the reduction of stress and cognitive burden during the use of VR and AR [7-9]. The results suggest that VR and AR can alleviate stress and cognitive load for users. These applications have been used to clarify anatomical conditions and facilitate the learning of practical tasks, thus reducing stress by providing presurgical training. Building on these findings, we hypothesized that in this study, using AR and VR simulations would be less taxing for participants when preparing for surgery, compared with the traditional method of using classical radiological images such as magnetic resonance imaging (MRI), CT, and X-rays.

Ghani et al [10] demonstrated that a passive oddball condition can be used to objectively assess mental workload through participants’ electroencephalography (EEG) [10]. By combining subjective questionnaires with the objective evaluation of workload via EEG, we hypothesize that in this study, the objective results on mental workload will align with the subjective data from the NASA-TLX questionnaire [11].

Bernhardt et al [6], Nicolau et al [12], and Dixon et al [13] described significantly improved visualization capabilities through the use of AR and VR, particularly in minimally invasive procedures. Based on these findings, we hypothesize that AR and VR will facilitate mental visualization of anatomical conditions more effectively than traditional 2D images, due to their 3D displays, ultimately leading to better outcomes, such as improved surgical performance.

To test the following 3 hypotheses, a passive oddball experiment with continuous EEG monitoring was conducted with 9 visceral surgeons as participants. They performed mock preoperative planning for the resection of a cancerous liver tumor using AR and VR headsets, as well as traditional radiological images (MRI and computed tomography scans, resolution: 5 mm; arterial and venous phases available) displayed on a standard computer screen.

- Hypothesis 1: AR and VR simulations will reduce mental workload compared with traditional radiological images.
- Hypothesis 2: The mental workload measured objectively using EEG will match with subjective reports from the NASA-TLX questionnaire.
- Hypothesis 3: AR and VR use will improve anatomical visualization and surgical performance compared with traditional 2D imaging.

To successfully test hypothesis 1, we are using 2 real, anonymized surgical cases that have been adapted for display in traditional radiological, VR, and AR environments.

To evaluate hypothesis 2, a state-of-the-art mobile EEG setup is used on the same cases, after which participants are asked to complete a commonly used, validated questionnaire on workload strain.

To evaluate performance in hypothesis 3, participants were required to assess the resectability of the tumor in each case, considering both its size and location to make an informed decision regarding surgical feasibility.

These hypotheses require both objective and subjective workload assessments, along with a comparative evaluation of task performance under different imaging conditions.

The design of the EEG task follows a standard passive auditory oddball paradigm, in which both a frequent and a rare auditory stimulus are presented. The EEG response to the target stimulus is analyzed, as it is expected to produce 2 distinct potentials: in addition to the early neuronal responses to the auditory stimulus (P1, N1, P2, and N2, collectively referred to as auditory evoked potentials [AEP]), a later response to the oddball modality—that is, the shift from a frequent to a rare stimulus—is expected to manifest as the P3 potential (hereafter referred to as event-related potential [ERP]). While the early AEPs are associated with basic neuronal processing, primarily reflecting the activity of working memory, the later ERPs are linked to more complex, higher-order cognitive processing of the presented stimulus [14].

The degree of these potentials is used to draw conclusions about the objective workload, specifically in terms of the attention required for a primary task. While the auditory stimuli are presented passively in the background, participants focus actively on a primary task—in our case, planning a surgery in different modalities. It has been demonstrated multiple times that EEG potentials are more pronounced when the primary task is less demanding, that is, requiring fewer neuronal resources: when the primary task is not resource-intensive, more mental resources are allocated to the passive background task, leading to higher EEG potentials for both AEPs and ERPs. If the primary task is more complex, fewer mental resources are allocated to the secondary, passive background task, resulting in less pronounced EEG potentials [10,14].

The objective workload in the 3 different conditions will be compared with the subjective workload measured using the NASA-TLX questionnaire. Additionally, the surgeons will be asked to perform simple tasks during the surgical planning process, such as measuring the diameter of lesions, to assess the quality of their work.

## Methods

### Ethical Considerations

All participants provided informed consent before the study. Participation was voluntary, and no form of compensation was offered. The study was approved by the Medical Ethics Committee (reference number 2020-150), the Data Protection and Information Security Management Unit at the University of Oldenburg, and the staff council at PIUS-Hospital. All participants were recruited through word of mouth by the medical staff of the University of Oldenburg’s Department of Visceral Surgery, located at PIUS-Hospital, Oldenburg. The study was conducted in accordance with the Declaration of Helsinki and the guidelines of Good Clinical Practice. Data were treated confidentially at all times. The database, containing anonymized patient data, was stored on a password-protected computer, ensuring access to authorized personnel only. Data were stored in a pseudonymized manner, with each participant assigned a numerical code.

### Participants

Nine participants with prior experience in liver surgery were recruited for this study. Although the sample size was relatively small, all participants were carefully selected from a well-defined target group—surgeons with experience in liver surgery—highlighting the study’s quality. Inclusion criteria included attending status (or residents in their fourth year of training), signed informed consent, no significant coffee consumption, and no history of neurological damage. Exclusion criteria for participants included excessive caffeine consumption, the use of psychoactive drugs or medications, and any mental or neurological disorders. These criteria were assessed in advance using a standard questionnaire for EEG measurements.

All participants were male, right-handed individuals with an average age of 36.8 (SD 5.59) years. Six of the participating surgeons were attending surgeons (including 2 senior surgeons), and 3 were residents in the later stages of their training. On average, before the study, participants had assisted in an average of 43 (SD 65.63) liver surgeries and had led an average of 16 (SD 29.7) surgeries.

### Stimuli

The acoustic oddball stimuli consisted of 2 sinusoidal tone pips, each with a duration of 50 ms and a rise/fall time of 5 ms. The standard tone, with a frequency of 500 Hz, had a probability of 88%, while the rarer target tone, at a frequency of 1 kHz, had a probability of 12%. Both tones were presented via loudspeakers (iLoud MTM; IK Multimedia), connected to an amplifier (the t.amp E4-130; Thomann), at a volume of 75 dB sound pressure level, with an interstimulus interval of 1 second. The tones were randomized with the constraint that a target tone was always followed by a standard tone. The stimuli were similar to those used by Williams et al [15]. The auditory stimuli were digitally created using MATLAB (version R2021a; The MathWorks, Inc.) and presented using Presentation Software (version 23.1; Neurobehavioral Systems).

### Conditions

Three different conditions were tested to assess their impact on focused attention during liver surgery planning. These conditions were (1) an AR headset, (2) a VR headset, and (3) a PC with traditional radiological images. The VR setup included the VIVE Pro headset (HTC Corporation), 1 controller, and 2 tracking devices, known as lighthouses. For AR, the Microsoft HoloLens 2 was used (Microsoft Corp.). The software used for visualization in VR was a custom-built setup previously used in our department [16]. For the AR setup, a demonstrator developed as part of this project was used. Traditional radiological images were displayed using the Xero Viewer (Agfa Health care) on a conventional PC. Screenshots of the Xero Viewer software and videos of the VR and AR conditions are available in Multimedia Appendices 1-4, respectively. In the AR and VR conditions, participants were presented with previously segmented 3D models in combination with radiological images. In the PC condition, participants were only presented with the radiological images. In all 3 conditions, participants were instructed to plan a liver resection using all available functions specific to each condition. In the AR and VR conditions, participants could draw on the 3D model, set markers, and manually measure anatomical structures. In the VR condition, additional functionalities were available, such as volume cropping and the placement of vascular clips. In both the AR and VR conditions, participants could also view or superimpose the corresponding MRI images onto the 3D model. The control mechanisms differed between the 2 conditions due to distinct hardware setups: the VR condition was controlled using dedicated controllers, while the AR condition relied on gesture-based interaction. In all 3 conditions, participants were asked to assess the size and segment of the largest tumor, as well as the resectability of the tumor. The primary goal was to keep participants engaged for at least 3 minutes in each condition, using a basic task (ie, diagnosing based on the images). This task was consistent across all conditions, regardless of the specific interface or available functionalities. Evaluating the usability of the respective software and its functionalities was beyond the scope of this study, particularly because both the AR and VR setups were custom-built and still under development.

### Experimental Setup

**Figure 1:**
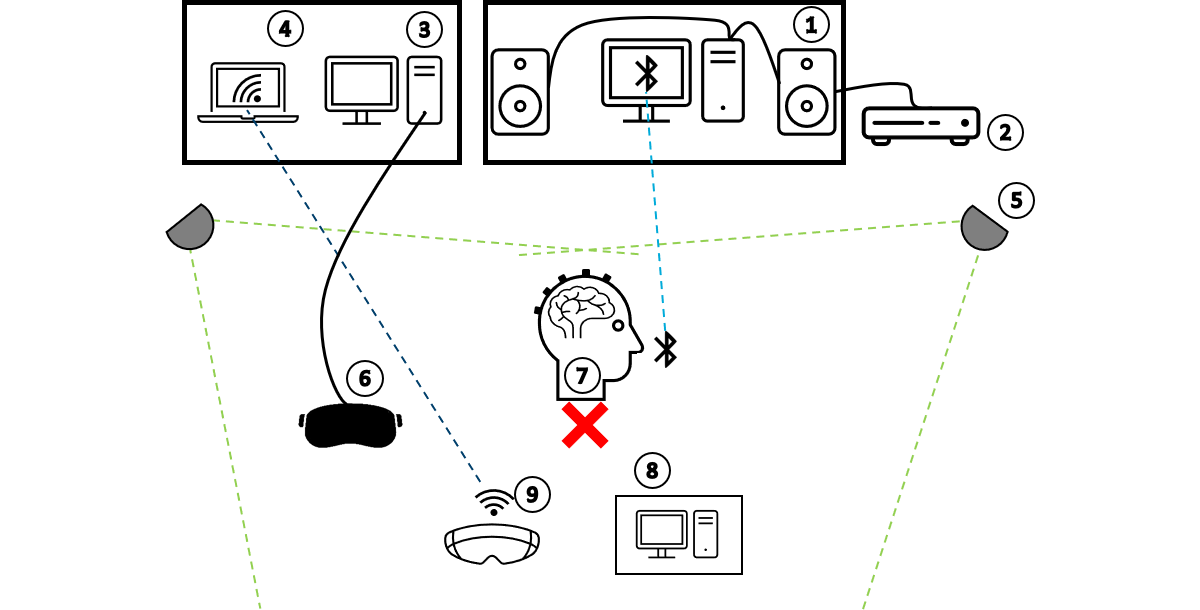
Experimental setup. 1: main computer for recording electroencephalography (EEG); 2: amplifier; 3: computer virtual reality (VR); 4: computer augmented reality (AR); 5: lighthouses for the VR headset; 6: VR headset; 7: EEG on marked position; 8: computer for conventional surgical planning; and 9: AR headset.

A mobile EEG amplifier (Smarting mobile; mbt) was connected to a standard 10/20 EEG cap (EasyCap GmbH) and recorded using software (Smarting Streamer v3.4.2; mbt) on a desktop computer running Windows 10 (Microsoft Corp.). The 2 speakers were placed next to the recording computer, with participants positioned 2 m in front of the speakers. During the experiment, care was taken to position the participants consistently in the same marked location across all 3 conditions to ensure that the stimuli were presented at the same sound level in each condition. A laptop was used to connect wirelessly to the AR glasses via Miracast, allowing the instructor to monitor what the participants were doing and seeing. The laptop was placed out of sight of the participants to avoid distractions. A second desktop computer was used to run the VR setup, with the VR headset connected to this computer and the 2 VR lighthouses mounted on tripods, enabling the participants to move freely while wearing the VR headset. To ensure that participants remained in the same position during all 3 conditions, the traditional radiological images presented in the PC condition were displayed on a computer connected to the hospital information system. This computer was placed on a mobile, height-adjustable table, with the screen positioned at eye level. This setup was designed to prevent the EEG cap from slipping due to excessive head movement and to allow participants to perform all conditions while standing (Figure 1). Controllers were used to operate the VR condition and were placed in the participants’ hands. In the AR condition, control was achieved through gesture-based interaction using the participants’ hands. No standardized training was provided before the experiment. However, if the conditions were unfamiliar to the participants or if they had any questions, a test run was conducted in which they could familiarize themselves with the controls and functions until they felt confident in handling the technology.

### Procedure

**Figure 2:**
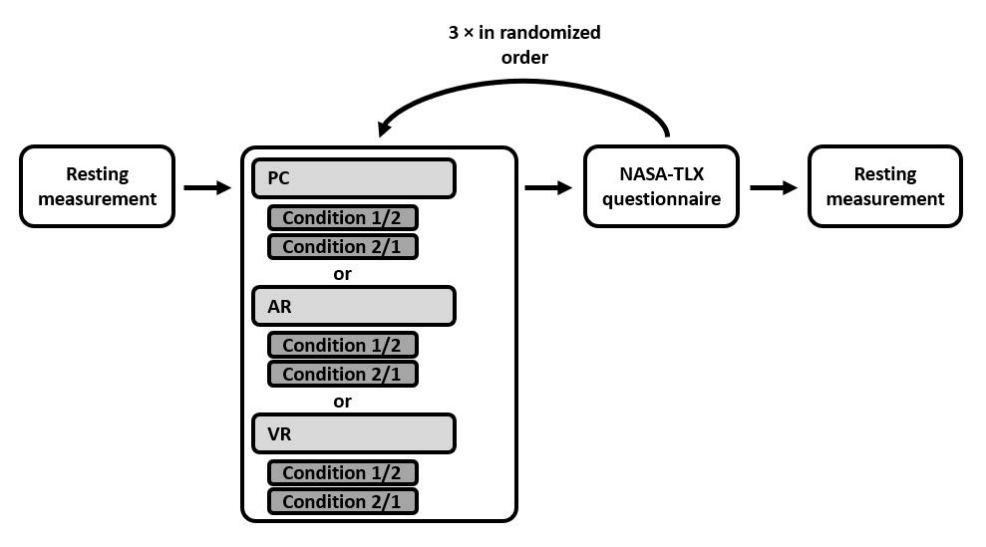
Experimental procedure flowchart: randomized order of conditions. AR: augmented reality; NASA-TLX: National Aeronautics and Space Administration-Task Load Index; VR: virtual reality.

Mobile EEG recordings (sampling rate: 250 Hz, Smarting Mobi; mbt) were obtained using a standard EasyCap EEG recording cap with 24 electrodes: Fp1, Fp2, AFz, F7, F3, Fz, F4, F8, T7, C3, Cz, C4, T8, CPz, M1, M2, P7, P3, Pz, P4, P8, POz, O1, and O2. The reference electrode was placed at a fronto-central position. Continuous EEG data were stored in .xdf format before being processed for analysis. For AEP analysis, EEG data were analyzed using EEGlab (V2020.0, open-source toolbox for MATLAB R2020a; MathWorks) on a standard PC running Windows 10 (Microsoft Corp.). For ERP analysis, EEG data were first converted to Brain Vision Analyzer format using MATLAB and then analyzed using Brain Vision Analyzer Software (version 2.2.1.8266, Professional Edition; Brain Products GmbH). EEG electrode impedances were adjusted to below 10 kΩ using 70% rubbing alcohol and abrasive conductive gel (EasyCap Abalyte Hi-Cl), following the manufacturer’s recommended protocol. After the electrodes were properly placed, 2 minutes of resting-state EEG was recorded with the eyes open and eyes closed before actual testing began. For the testing, 1 of the 3 conditions—“AR,” “VR,” or “radiological images”—was selected in randomized order. In each of the 3 conditions, participants were required to plan 2 different surgeries, which included measuring the dimensions of the tumors and identifying their anatomical locations within the liver to determine whether the tumors were resectable. The 2 patient cases were presented in randomized order (see Figure 2 for the experimental procedure). By the end of the session, each participant had completed 6 sets of surgical planning (2 cases for each of the 3 conditions). The task for the participants was to plan a surgical procedure to remove cancerous tissue from the liver. The processing time for each condition (AR, VR, or radiology) was 10 minutes, with 5 minutes allocated to each patient case. Throughout the task, auditory stimuli were presented to the participants via 2 speakers in the background. Before switching to the next condition, the participants’ subjective workload was assessed using the NASA-TLX questionnaire.

### NASA-TLX

The NASA-TLX workload questionnaire has been validated as an effective measure of subjective workload in medical work environments [17].

The NASA-TLX is a widely used tool for assessing subjective workload. Originally developed for aviation settings by NASA, it has since been adapted for use in various fields, including health care [17]. The NASA-TLX measures 6 dimensions of workload: mental demand, physical demand, temporal demand, effort, frustration, and perceived performance. Each dimension is rated on a scale from 0 (low) to 100 (high) [18].

### Data Preprocessing of EEG Data

To retain the frequency components relevant for EEG analysis, the recordings were filtered using a bandpass filter (low cutoff: 0.05 Hz, high cutoff: 30 Hz, and filter order: 4). A notch filter was also applied at 50 Hz to remove power line interference. The continuous EEG data were divided into 1000 ms epochs, with 200 ms before and 800 ms after the stimulus. The data were then categorized into 2 categories: standard stimulus (nontarget: 0.5 kHz) and target stimulus (1 kHz). Automatic artifact rejection was performed by discarding all epochs where fluctuations greater than +100 µV or –100 µV occurred within 100 ms. Additionally, all epochs underwent manual visual inspection, focusing on signals from electrodes FP1 and FP2 to identify strong artifacts due to eye movements. Averages were then calculated for the epochs recorded during the presentation of the target stimulus in each of the 3 conditions: 1 average for the EEG response to the target stimulus during AR, 1 for the EEG response during VR, and 1 for the EEG response during surgical planning using traditional radiological images. Averages were calculated for each participant to facilitate further statistical analyses. Baseline correction was applied to prevent potential linear drifts in the recorded signals and to ensure that the grand averages across participants and conditions were comparable. For AEP analysis, the data were re-referenced to electrodes T5 and T6, while for ERP analysis, the data remained referenced to the physical fronto-central reference.

## Results

### Subjective Workload

**Figure 3:**
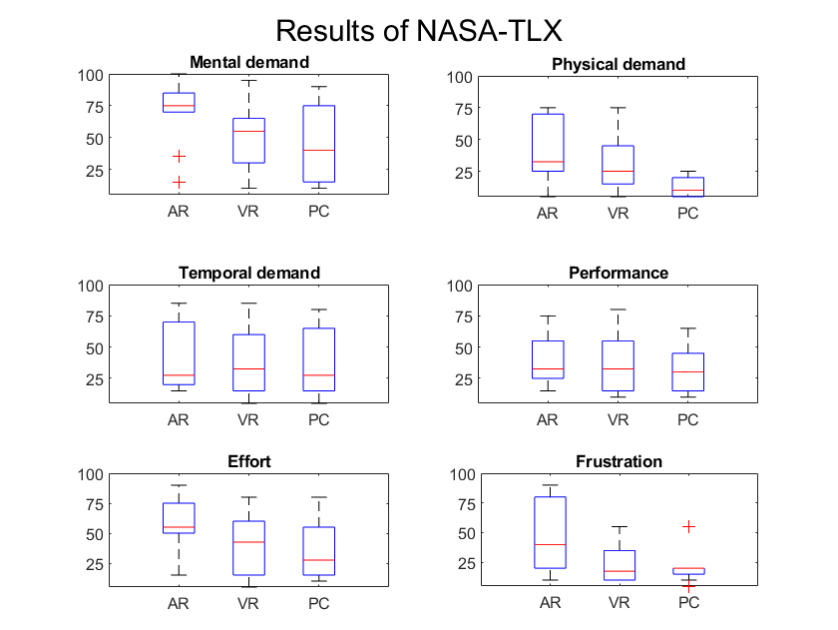
National Aeronautics and Space Administration-Task Load Index (NASA-TLX) scores. AR: augmented reality; VR: virtual reality.

The median physical demand score for the AR condition was 30 (IQR 25-70) on the NASA-TLX scale, higher than the scores for the VR (median 25, IQR 15-45) and PC (median 10, IQR 5-20) conditions. For the perceived performance and temporal demand categories, all 3 conditions yielded the same NASA-TLX scores: 25 for temporal demand and 30 for perceived performance. Additionally, for the mental demand, effort, and frustration categories, the AR condition showed the highest median scores: 75 (IQR 70-85) for mental demand, 55 (IQR 50-75) for effort, and 40 (20-80) for frustration (Figure 3).

Examination of the box plots reveals that the mental demand score for the AR headset (median 75, IQR 70-85) was higher than those for both the VR headset (median 55, IQR 30-65) and the PC (median 40, IQR 15-75) conditions. Similarly, higher scores for effort and frustration were observed in the NASA-TLX evaluations when using the AR headset (median 55, IQR 50-75 for effort; and median 40, IQR 20-80 for frustration) compared with using the VR headset (median 40, IQR 15-60 for effort; and median 15, IQR 10-35 for frustration) or the PC condition (median 25, IQR 15-55 for effort; and median 20, IQR 15-20 for frustration). In terms of physical demand, the score was lower in the PC condition (median 10, IQR 5-20) compared with the AR (median 30, IQR 25-70) or VR (median 25, IQR 15-45) condition.

A Friedman test was performed to assess significance (significance level of .05), as the data did not follow a normal distribution according to the Kolmogorov-Smirnov test. To account for multiple comparisons, the Benjamini-Hochberg method was applied for correction using a web-based calculator [19]. Assuming a 95% CI, no significant differences (*P*=.10) were found.

### Electrophysiological Recordings—Early Components

**Figure 4:**
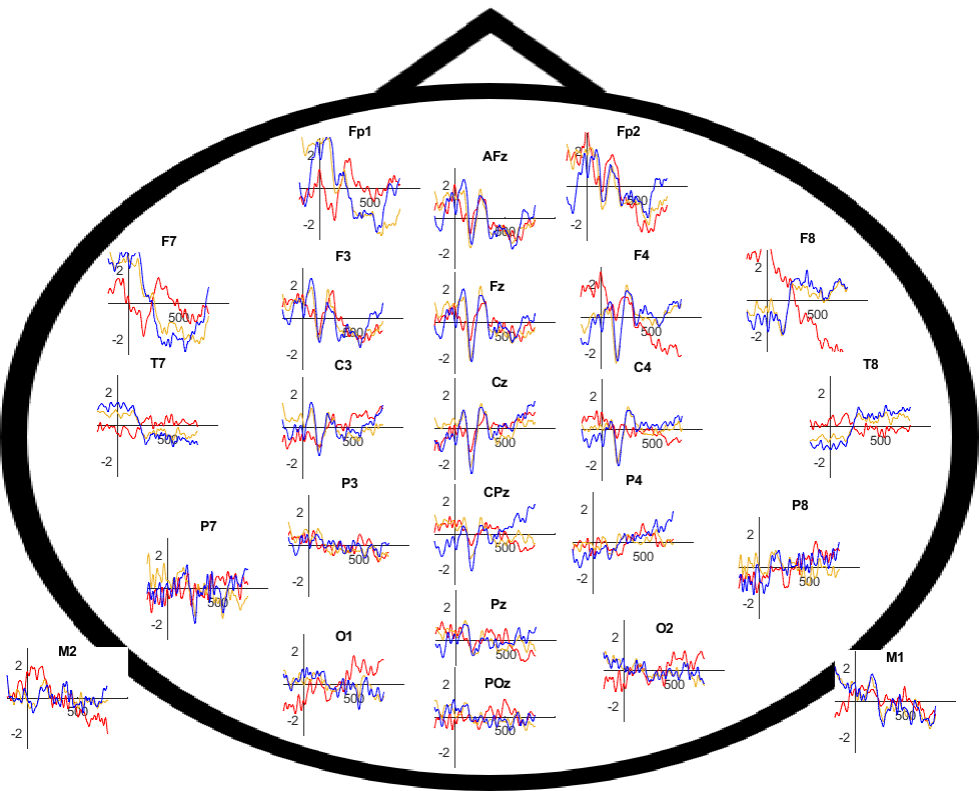
Event-related potentials as response to the target tone for all electrodes and all 3 conditions.

AEPs were primarily observed on the electrodes along the central row and midline (AFz to Pz, F3/C3, and F4/P4). Grand averages were calculated for all electrodes (Figure 4).

**Figure 5:**
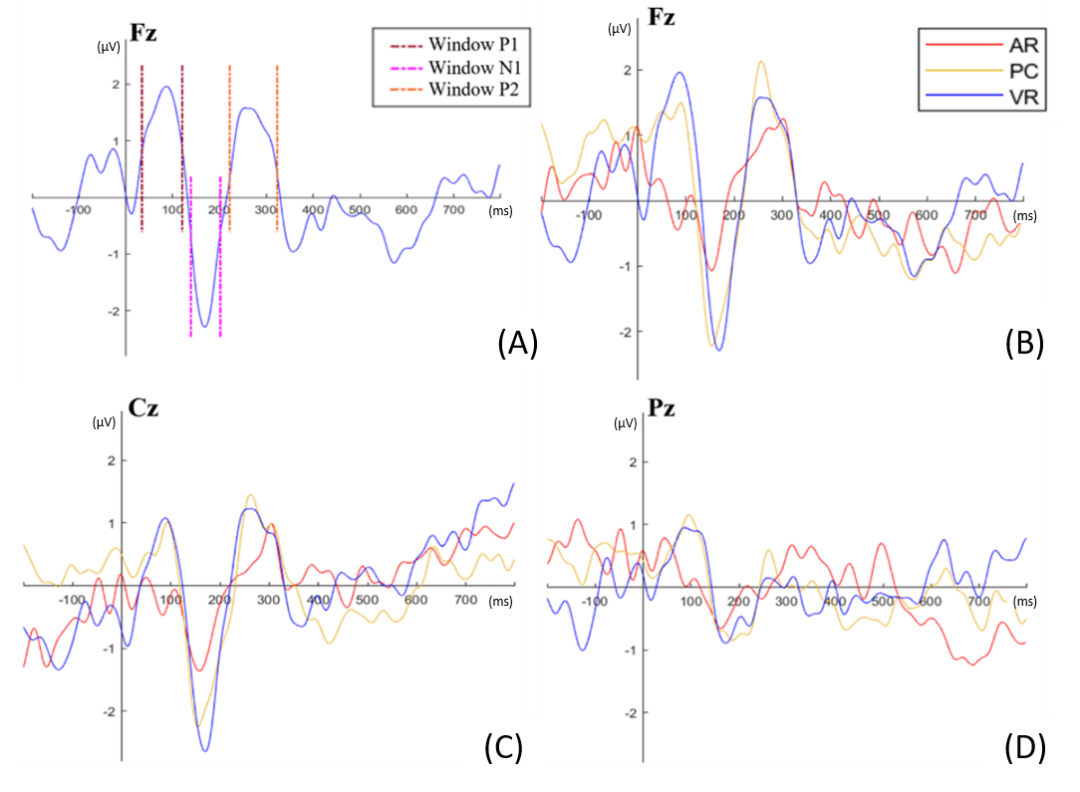
Grand averages of event-related potentials as a response to the target tone for statistically analyzed electrodes under all 3 conditions: (A) temporal windows for the event-related components P1, N1, and P2, exemplarily shown for the electrode position Fz; (B) grand averages of the electrode Fz; (C) grand averages of the electrode Cz; (D) grand averages of the electrode Pz. AR: augmented reality; VR: virtual reality.

To statistically analyze the amplitudes for each participant, the peaks and their corresponding latencies were identified for all conditions at the central electrodes Fz, Cz, and Pz (Figure 5B-D). To statistically verify differences in the amplitudes of the grand averages, the approach outlined by Handy [20] was used as a guide. The intervals for P1, N1, and P2 were marked accordingly (Figure 5A). The resulting peaks and latencies for each participant were then analyzed using a repeated measures analysis of variance (rmANOVA) in SPSS (version 28.0.1.0; IBM Corp.) with the factors patient case, condition, electrode, and ERP components. A significant difference was observed between the ERP components (*F*_1.718,13.743_=46.754, *P*<.001).

The tendencies observed in the grand averages were not supported by the statistical analysis, as no significant differences were found between patient cases (*P*=.57), conditions (*P*=.71), or electrodes (*P*=.49). To determine the latencies of the AEP components (P1, N1, and P2), the method described by Handy [20] was also applied. The latency of the identified peaks within the defined intervals was measured and recorded for each participant. Subsequently, an rmANOVA was conducted with the factors patient case, condition, electrode, and ERP components to identify significant differences in the temporal occurrence of these components.

The tests of within-subject effects revealed a significant interaction among condition, electrode, and ERP components (*F*_3.401,27.211_=6.747, *P*=.001). The results of the post hoc pairwise comparisons are presented in Table 1. According to these comparisons, the P1 component at the Fz electrode occurs significantly (*P*=.01) earlier in the VR condition (mean 75.3 ms, SD 25.8 ms) compared with when using traditional radiological images (mean 99.4 ms, SD 28.6 ms). The Cz electrode also demonstrated a significant (*P*=.04) difference in the P1 component between the VR condition (mean 111.1 ms, SD 9.9 ms) and the AR condition (mean 80.4 ms, SD 32.1 ms). Additionally, the P2 component in the AR condition (mean 290.6 ms, SD 23.5 ms) occurred significantly (*P*=.03) later than in the traditional radiological images condition (mean 265.3 ms, SD 13.1 ms). Significant differences were also observed for the P1 component at the Pz electrode, with the VR condition (mean 62.8 ms, SD 24.8 ms) occurring significantly earlier (*P*=.04) than both the AR condition (mean 89.1 ms, SD 14.6 ms) and the traditional radiological images condition (mean 92.8 ms, SD 28.4 ms; *P*=.04). No significant (*P*=*.*12) interactions were found for the N2 component.

**Table 1:** Significant results of the post hoc tests (pairwise comparisons).

Electrode and ERP^a^ component			Condition (I)		Condition (J)		Latency (ms), mean				Difference between means (I–J)		*P* value
							Condition (I)		Condition (J)				
**Fz**													
	P1	VR^b^		PC^c^		75.3		99.4		–24.1		.01	
**Cz**													
	P1	VR		AR^d^		111.11		80.4		30.71		.04	
	P2	AR		PC		280.7		265.3		25.4		.03	
**Pz**													
	P1	VR		AR		62.9		89.1		–26.2		.04	
				PC		62.9		92.9		–30		.04	

^a^ERP: event-related potential.

^b^VR: surgery planning using the virtual reality setup.

^c^PC: surgery planning using traditional radiological images on a desktop computer.

^d^AR: surgery planning using the augmented reality setup.

### Electrophysiological Recordings (P3)

**Figure 6:**
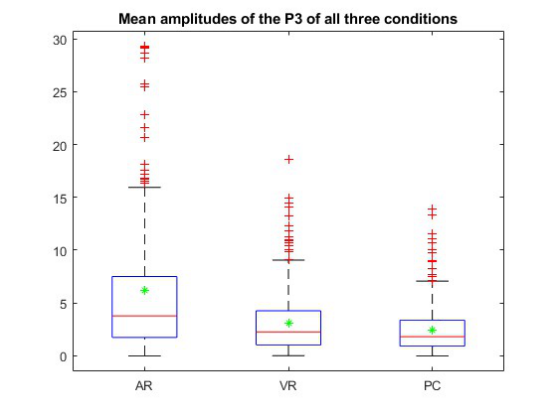
Amplitude of the P3 component of the event-related potential across all 3 conditions: the augmented reality (AR) approach (left), which showed the highest P3 amplitude (mean 6.20 µV, SD 6.84 µV); the virtual reality (VR) condition (center), with a mean amplitude of 3.08 µV (SD 2.80 µV); and the classical approach (right), which exhibited the lowest P3 amplitude (mean 2.45 µV, SD 2.26 µV). No statistically significant differences were found (*F*_(1.94,10.58)_=1.469; *P*=.26).

For the P3 component of the ERP, peak amplitudes within the time range of interest (300-400 ms poststimulus) were calculated. A grand average for all 3 conditions (radiology, VR, and AR) was computed using data from all electrodes (Figure 6). A clear trend was observed, with the classical radiological approach eliciting the lowest P3 amplitude (mean 2.45 µV, SD 2.26 µV), VR eliciting the second largest P3 amplitude (mean 3.08 µV, SD 2.80 µV), and the AR condition eliciting the highest P3 amplitude (mean 6.20 µV, SD 6.84 µV). SDs were high, and no statistical significance was found using an rmANOVA in SPSS (version 28.0.1.0) with the factors patient case, condition, and P3 amplitude (*F*_1.94,10.58_=1.469, *P*=.26).

### Post Hoc Power Analysis

Given the small sample size (N=9), a post hoc power analysis was conducted to determine the effect size and assess whether a larger sample might yield different results. Partial eta-squared (η^2^=0.114) was calculated for the interaction between experimental conditions using SPSS. To estimate the sample size required for significant results, a web-based calculator was utilized [19], indicating that a sample size of 101 participants would be needed to achieve a statistical power of 0.8 at a significance level of α=.05.

### Correlation

A correlation analysis was conducted between the N1 component, which demonstrates a decreased amplitude with increased cognitive workload [10], and the “Mental Demand” and “Effort” dimensions of the NASA-TLX questionnaire. The results revealed a correlation coefficient of *r*=–0.0042 between the N1 component and “Mental Demand,” and *r*=0.066 between the N1 component and “Effort.”

### Performance

All participants correctly identified the resectability of the tumors in all conditions and for every patient case, based on their accurate assessment of tumor size and localization.

## Discussion

### Findings and Implications

The aim of this study was to establish a method for objectively determining the additional workload generated using AR or VR glasses in a clinical context for the first time. For this purpose, 2 runs of planning a liver surgery were performed under each of the 3 conditions. In the background, a passive oddball experiment was conducted to produce clear early AEP components (P1, N1, and P2) as well as the P3 component of the ERP in the EEG recordings, guided by the work of Ghani et al [10].

### Subjective Workload

NASA-TLX scores indicate a tendency toward higher scores, suggesting a greater subjective workload for AR planning, particularly in terms of “mental demands” and “frustration.” However, this trend does not reach statistical significance when compared with VR or the traditional approach using radiological images, likely due to the small sample size. Moreover, the large variances observed in the data could have contributed to the lack of significant findings, as they indicate substantial variability in subjective workload perceptions among participants. The observed tendencies may be attributed to the more complex and unfamiliar gesture controls of the AR headset. In addition, a poor internet connection may have resulted in higher latencies, potentially affecting gesture control and thus leading to higher workloads when using the AR demonstrator.

Higher physical demands were expected in the AR and VR setups compared with the traditional approach. A tendency was observed in the subjective ratings, as using a stationary mouse on a desktop PC required significantly less physical effort compared with using an AR or VR headset.

### Objective Workload

The expectation that using an AR or VR setup would generally make planning surgery easier due to its 3D projection of the anatomical situation was not confirmed.

EEG data indicate a trend with more pronounced early AEPs when using VR and classical radiological images compared with AR, suggesting that AR results in the highest workload for the surgeon, while VR does not increase workload compared with classical radiological images. Differences are especially visible in the ERPs P1 and N1 components: no P1 component was found for the AR condition, whereas the N1 component showed a significant difference for the AR condition compared with radiological images and VR on the level of grand averages. This aligns with the finding of Ghani et al [10], indicating that the N1 component becomes less pronounced with increased task difficulty due to the more complicated gesture control of the AR headset and limited opportunity to familiarize with the gestures required to handle 3D objects in AR.

We would like to emphasize that the significance at the electrode Pz should be treated with caution, as the amplitudes here are generally lower. The results for the electrode Pz are clearly noisier, likely due to its position being obstructed by the fastening of the glasses, which are attached to the head near the position of electrode Pz. These results should therefore be interpreted with caution.

When examining the significances of the latencies of the different early AEP components, it is noticeable that there are occasional significances that do not follow a coherent pattern.

Concerning the event-related P3 wave in response to the target stimulus of the passive oddball paradigm, the pattern found differs from that seen in the early potentials: a trend from a low P3 in the radiology condition to a high P3 in the AR condition can be observed. The studies conducted by Thees et al [7], Küçük et al [8], and Chao et al [9], which asserted that AR and VR headsets result in reduced cognitive load, can thus only be partly substantiated in this investigation.

Based on our data, we conclude that the use of the passive oddball paradigm is a suitable tool to measure the objective workload between the radiology, AR, and VR setups in planning a complex surgery. There appear to be measurable differences in the neuronal processing of 2D screen images when compared with VR and AR images. However, the answer to why the different modalities of EEG components—early AEP versus ERP—yielded different findings is complex and likely rooted in distinct, complex neuronal and psychological processes responsible for producing these potentials. To better understand the different psychological processes behind the processing of 2D screen images versus VR and AR images, in addition to increasing the number of participants, a more in-depth analysis of the P3 potential is needed. In our analysis, we only looked at the grand average of the peak amplitude. However, it is also important to investigate the location of the most pronounced P3 potentials, as well as the dynamics—that is, the distribution of P3 peak latencies across different electrode localizations and how this distribution changes over time. Current research indicates that the P3 potential arises as a combination of different subcomponents, P3a and P3b, which may be involved in distinct neuropsychological processes. This differentiation needs to be addressed in further studies.

A simple but feasible explanation for the different findings between early AEPs and P3-ERPs could be attributed to the different mental processes involved: while the complex cognitive processing of AR pictures, represented by the P3 potential, might be less demanding for the user, aligning with findings from Thees et al [7], Küçük et al [8], and Chao et al [9], the difficult or less familiar navigation in the AR—and to a lesser extent, VR—environment may lead to higher demands on basic neuronal tasks, such as visuomotor coordination, which are more strongly represented by early neurological potentials than by late cognitive potentials. While this remains speculative at this point, we believe that our study should be regarded as a proof-of-principle, demonstrating a foundation for further research with a larger sample size and in more complex settings. This will allow for a deeper investigation into this issue.

The lack of a significant correlation between the N1 component and the subjective measures of mental demand and effort is likely influenced by the extremely small sample size in this study. However, this result also suggests that cognitive workload, as measured by physiological markers such as EEG, may not necessarily align with the subjectively perceived workload. This finding underscores the importance of using objective measures in assessing workplace stress and cognitive load. Relying solely on subjective assessments may not fully capture the cognitive demands placed on individuals, highlighting the need for more robust and comprehensive approaches in future research.

The use of a mobile EEG was particularly useful in this study. The high degree of mobility provided by the use of portable smartphones as recording devices allowed for recordings to be made in situations closely aligned with the operation without restricting participants’ freedom of action. The validity of corresponding mobile EEGs has been previously described by Wascher et al [21]. Moreover, the work of El Basbasse et al [22], among others, has confirmed that reliable data can be derived using mobile EEG recordings, including the implementation of the EasyCap system in conjunction with VR headsets [21,22]. The results of this study support this research. Additionally, our findings demonstrate that the study design, when combined with mobile EEG technology, enables the investigation of the additional workload induced by various medical and clinical devices. Notably, even head-worn devices cannot be unequivocally excluded from this measurement approach.

The AR setup was perceived and measured as the most difficult of the 3 setups. As mentioned, this may be due to the complexity of its controls. While the classical approach was well-known to the participants, controlling the VR setup appeared to be more intuitive than using the AR setup. However, it is crucial to consider that AR headsets represent a relatively nascent technology, and gesture recognition is still in its developmental stages, with anticipated advancements in future iterations of AR headsets. It appeared that participants were overwhelmed by handling the headset without a controller or corresponding control element, as gesture control was a new experience for most of them. This difficulty may be overcome by more accurate training of users before working with the AR setup. We believe that our work could be used as a tool to assess how well users are trained in using the AR setup and may be used to generally investigate the need for training when navigating in an AR and VR environment, even beyond its medical applications. Additionally, more complex, subconscious explanations could be considered: The VR approach might benefit from the possibility of exclusively displaying the anatomical model, while the AR setup might strain participants’ mental capabilities more due to the simultaneous display of the computer-generated 3D model along with the real-world environment. Furthermore, factors such as the different displays of the HoloLens and the VIVE Pro could contribute to the differences in participants’ workload. However, this requires more detailed investigations. It must be considered in this discussion that the 2D viewing habits trained over decades are completely abandoned. New 3D viewing habits must be relearned in AR and VR, along with the integration of voice and gesture control.

This study makes a significant contribution to the existing body of research by specifically focusing on the mental workload of medical professionals. Unlike the studies listed in Table 2, which mainly explore cognitive load in nonmedical settings or involve participants lacking domain-specific expertise, our research is uniquely positioned within the context of clinical practice. By using objective EEG measurements, we were able to assess workplace strain in real-world scenarios, providing insights into the cognitive demands experienced by surgeons when using AR and VR technologies. This focus on medical professionals, coupled with the use of advanced neuroergonomic methods, highlights the practical relevance and novelty of our findings. Our study not only expands the understanding of how AR and VR technologies impact mental workload in high-stakes environments but also provides valuable data that could inform the optimization of these technologies for clinical use.

**Table 2:** Comparison of recent research studies investigating mental workload using EEG^a^ measurements in AR^b^ and VR^c^ applications.

Study	Population	EEG application	Key findings
Alessa et al [23]	28 Healthy males (mean age 32.12 years)	EEG power analysis	AR instructions reduced task time compared with paper instructions, particularly for high-demand tasks. AR increased mental workload and information processing (germane load).
Binias et al [24]	Pilots in short-haul flight simulator sessions	EEG power analysis	Positive correlation between theta power (frontal lobe) and reaction time. Significant event-related changes in the frontal lobe for theta and beta waves.
Ji et al [25]	Pilots in VR-simulated missions	Focusing on β rhythms	β rhythm changes correlated with high-difficulty tasks. A significant relationship between mental workload and β rhythms.
Aksoy et al [26]	General population	Visual event–related potentials such as N1, P1, and P3	ERP^d^ waveforms were similar between VR HMD^e^ and CS^f^ P3 amplitudes were comparable between VR HMD and CS. N1 amplitudes were significantly higher in VR HMD compared with CS. P1 amplitudes were higher at the occipital region in VR HMD.
Giorgi et al [27]	General population	Global field power, alpha, and theta	A higher Workload Index was observed in the museum compared with VR. Overall, the museum showed higher engagement potential, while VR potentially supported greater embodiment.
Marucci et al [28]	General population	P300 potentials with respect to the unimodal (visual) and visual and tactile bimodal stimulation	Multisensory stimuli significantly improved performance and reduced EEG-based workload only in high load conditions. Multimodal stimulation (visual-audio-tactile) decreased latency and increased P300 amplitude, indicating faster and more effective stimulus processing.
Atici-Ulusu et al [29]	Employees on an automobile assembly line	EEG and NASA-TLX were used to measure cognitive load	Cognitive load (measured by EEG) was lower with AR glasses. NASA-TLX scores decreased by 10% when using AR glasses, indicating reduced cognitive load. No significant difference in cognitive load between the first and last days of AR glasses usage.
Trupti et al [30]	General population	EEG signals were analyzed using autoregressive modeling, fuzzy c-means clustering, hidden Markov model, and deep recurrent neural network classification	The proposed method for classifying cognitive workload using EEG signals achieved 97.8% accuracy. Handcrafted features combined with temporal dynamics modeling outperformed state-of-the-art methods. The optimal performance was achieved with 4 electrodes.
Luque et al [31]	General population	Event-related potentials such as P3 and contingent negative variation	VR effectively mimicked real-world cognitive load and decision-making processes. Significant ERPs related to attention and decision-making were elicited in VR.
Mondellini et al [32]	General population	Calculated the MWL index^h^	Different task complexity levels significantly affected MWL, increasing subjective measures and decreasing performance. HMD generally resulted in lower EEG-derived MWL, indicating reduced cognitive load for some tasks.

^a^EEG: electroencephalography.

^b^AR: augmented reality.

^c^VR: virtual reality.

^d^ERP: event-related potential.

^e^HMD: head-mounted display.

^f^CS: computer screen.

^g^NASA-TLX: National Aeronautics and Space Administration-Task Load Index.

^h^MWL: mental workload.

To provide an outlook beyond the scope of this study, we believe that AR and VR can be very useful technologies in the clinical context when used for the appropriate tasks. For instance, VR could be more suitable in a teaching setting or for preoperative planning, whereas AR holds immense potential beyond preoperative planning, particularly in the operating room. Its ability to provide an open view of the real environment while maintaining sterility allows for virtual overlays of anatomical structures on a patient’s body during surgery, enhancing visualization and accuracy. Whether the significant benefits of these technologies outweigh the potential additional workload for surgeons should be further investigated in future studies.

Furthermore, investigating the impact of training, the continuous development of software and hardware, and the increasing familiarity of the younger generation of physicians with these new technologies on measures of workload would be of great interest. Such research could provide valuable insights into the potential benefits and challenges posed by integrating advanced technologies such as AR and VR in medical practice. Further exploration in this area may also facilitate the design of tailored training programs and technological interventions to optimize the utilization of these innovations and improve overall health care outcomes.

### Limitations

Concerning our hypothesis that the use of AR or VR might improve surgical performance, no conclusive results were found. Basic tasks, such as deciding whether a tumor is resectable or not, were performed with the same quality across all experimental conditions. This might indicate that AR and VR technology does not inherently lead to better performance in trained surgeons for simple tasks, but this needs to be evaluated further. Future studies should include more specialized criteria for evaluating performance, particularly to assess finer aspects of clinical decision-making in AR and VR environments. This could involve more complex cases or tasks that better reflect the challenges faced in surgical planning and execution, allowing for a deeper understanding of how these technologies impact surgical performance and outcomes. This was beyond the aim of this study. However, based on our findings, we suggest that increased performance might be found in a more challenging task, but not concerning simple anatomical measurements.

On a more general outlook, one might speculate that increased workload over a prolonged period might eventually lead to increased fatigue, potentially decreasing a surgeon’s performance after an extended period. This is purely speculative and needs to be investigated in detail through further studies, possibly utilizing our suggested approach for workload measurement—correlating electrophysiological and performance results.

This study focused solely on the workload of the 3 conditions. It should be made clear that there are scenarios where a higher workload is acceptable when weighed against the benefits that such an approach would yield. While the VR setup might be suitable for surgical planning due to its lack of visibility into the real environment, the AR setup may be the more suitable approach during surgery—such as projecting radiological images as 3D models directly into the patient’s situs during a procedure to localize, for example, tumors or blood vessels. In these situations, however, technologies such as AR glasses can offer significant advantages due to their see-through capabilities, allowing a slightly higher workload, as observed in this study, to be acceptable if parameters such as patient safety are enhanced through the use of such technologies.

Interpretation of our results should be conducted with caution due to the very low number of participants (N=9), resulting in limited statistical power. This work should be seen as a feasibility study on how to measure objective workload while using AR or VR in a surgical simulation setting. However, we believe the study still provides valuable data, as experienced surgeons in a clinical setting used real-life patient cases with state-of-the-art equipment for AR and VR presentation.

### Conclusions

We aimed at developing a possible setup for objective measurement of workplace strain when using AR and VR technologies in the medical profession. We conclude that the use of a passive oddball paradigm is a feasible approach for this objective. The measurement of workload during surgical planning, and thereby likely also during other medical applications when using AR or VR technology, can be sufficiently done using an oddball paradigm. This approach proves to be a good opportunity to objectively determine workplace strain in a medical setting.

In future studies, the use of this objective measurement could potentially replace subjective questionnaires and significantly enhance the significance of the measured workplace strain. This approach, with the objectivity provided, may even become a commercially available application for medical education and surgical training, utilizing automated EEG analysis. Furthermore, we consider that AR or VR approaches to displaying radiological images are not inherently superior to the classical approach. The use of AR technology, in particular, seems to require a certain amount of training, likely due to the novelty of gesture control. However, we are confident that with future developments in AR technology and gesture recognition, these issues will become less significant over time. Regardless, the differences in workload between AR and VR highlight the need for intuitive user interaction and ease of use for any new technology, especially when applied in a medical setting.

Additionally, integrating machine learning techniques could indeed enhance the analysis of physiological data, allowing for the development of predictive models that may generalize across different tasks and populations. Such approaches could provide deeper insights into cognitive workload and potentially lead to the creation of real-time monitoring tools to optimize performance in high-stakes environments such as surgery. This could eventually result in commercially available solutions for use in medical education or surgical training.

In conclusion, this paper demonstrates the feasibility of utilizing a mobile EEG setup in conjunction with a passive oddball paradigm to assess the potential additional cognitive load imposed by emerging technologies such as AR and VR headsets in a clinical setting.

## Appendix

Multimedia Appendix 1 Screenshot of the PC condition.

Multimedia Appendix 2 Video part 1 of the virtual reality condition.

Multimedia Appendix 3 Video part 2 of the virtual reality condition.

Multimedia Appendix 4 Video of the augmented reality condition.

## Abbreviations

AEP: auditory evoked potential
AR: augmented reality
EEG: electroencephalography
ERP: event-related potential
MRI: magnetic resonance imaging
NASA-TLX: National Aeronautics and Space Administration-Task Load Index
rmANOVA: repeated measures analysis of variance
VR: virtual reality

## Data Availability

The data presented in this study are available on request from the corresponding author.
