# Supplementary figures and images for: Measuring Bound Attention During Complex Liver Surgery Planning: Feasibility Study
Source: JMIR Form Res. 2025 Jan 8;9:e62740. doi: 10.2196/62740 (PMC11754988; doi:10.2196/62740)

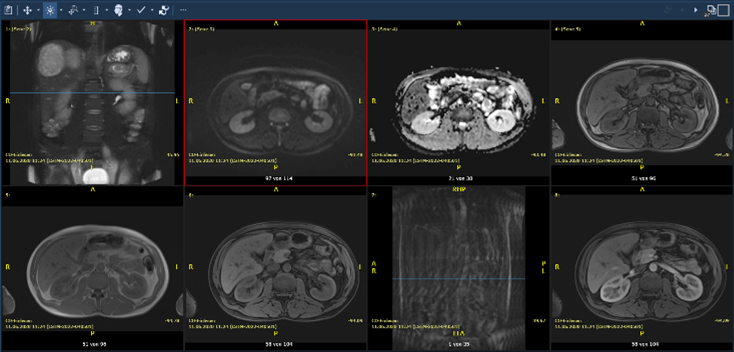

Supplement: Multimedia Appendix 1 [file formative_v9i1e62740_app1.png]
